# Melatonin for Neonatal Encephalopathy: From Bench to Bedside

**DOI:** 10.3390/ijms22115481

**Published:** 2021-05-22

**Authors:** Raymand Pang, Adnan Advic-Belltheus, Christopher Meehan, Daniel J. Fullen, Xavier Golay, Nicola J. Robertson

**Affiliations:** 1 Institute for Women’s Health, University College London, London WC1E 6HU, UK; r.pang@ucl.ac.uk (R.P.); a.advic-belltheus@ucl.ac.uk (A.A.-B.); chris.meehan@ucl.ac.uk (C.M.); 2 Translational Research Office, University College London, London W1T 7NF, UK; d.fullen@ucl.ac.uk; 3 Department of Brain Repair and Rehabilitation, Institute of Neurology, University College London, London WC1N 3BG, UK; x.golay@ucl.ac.uk; 4 Centre for Clinical Brain Sciences, University of Edinburgh, Edinburgh EH16 4SB, UK

**Keywords:** neonatal encephalopathy, melatonin, neuroprotection, therapeutic hypothermia, hypoxia-ischaemia

## Abstract

Neonatal encephalopathy is a leading cause of morbidity and mortality worldwide. Although therapeutic hypothermia (HT) is now standard practice in most neonatal intensive care units in high resource settings, some infants still develop long-term adverse neurological sequelae. In low resource settings, HT may not be safe or efficacious. Therefore, additional neuroprotective interventions are urgently needed. Melatonin’s diverse neuroprotective properties include antioxidant, anti-inflammatory, and anti-apoptotic effects. Its strong safety profile and compelling preclinical data suggests that melatonin is a promising agent to improve the outcomes of infants with NE. Over the past decade, the safety and efficacy of melatonin to augment HT has been studied in the neonatal piglet model of perinatal asphyxia. From this model, we have observed that the neuroprotective effects of melatonin are time-critical and dose dependent. Therapeutic melatonin levels are likely to be 15–30 mg/L and for optimal effect, these need to be achieved within the first 2–3 h after birth. This review summarises the neuroprotective properties of melatonin, the key findings from the piglet and other animal studies to date, and the challenges we face to translate melatonin from bench to bedside.

## 1. Introduction

Intrapartum hypoxic-ischemic (HI) events, resulting in neonatal encephalopathy (NE), lead to significant neonatal mortality and morbidity worldwide. In 2010, an estimated 1.15 million babies developed NE globally, with an incidence of 8.5 per 1000 live births, and almost 45% of infants with NE either died or developed moderate-severe neurodevelopmental impairment [1]. Furthermore, over the last 20 years, the global burden of disease resulting from NE on childhood disability has increased by 35% as a result of improved survival [2].

In high resource settings, the reported incidence of brain injury, secondary to HI, is between 1.5 and 3.5 per 1000 live births [3,4,5]. Therapeutic hypothermia (HT), reducing the core temperature of at-risk infants to 33.5 °C for 72 h, has become standard care for moderate-severe NE following suspected HI in high resource settings. Whilst HT is associated with a relative risk reduction of 25% in the composite outcome of death or major disability and a number needed to treat of 7–9, 46% of infants still developed adverse outcomes in clinical trials [6]. HT has expanded over the last decade to become an integral part of neonatal neurocritical care, however improvement in outcomes is modest [7,8]. Whilst mortality rates have reduced by more than half (10.9% in 2014–2016) since clinical trials [9], the rate of cerebral palsy remains at 19% [7,8] and even in its absence, 34% of children still develop neurocognitive and psychological impairment that may impact school performance [10]. Diffusion-weighted magnetic resonance imaging in school-aged children without cerebral palsy who were cooled at birth showed widespread defects in the white matter microstructure, with disruption in brain network connectivity over regions affecting visuo-spatial processing and attention [11]. Further neuroprotective interventions are needed; in preclinical [12,13,14] and clinical studies [7], attempts to exploit HT effects further, by increasing the depth and duration suggest that the current protocol of 72 h cooling to 33.5 °C is optimal [15]. Therefore, adjunct therapies to augment HT hold the greatest promise to improve outcomes in babies with NE.

The burden of NE secondary to intrapartum events is disproportionately higher amongst low- and middle-income countries (LMIC). In particular, sub-Saharan Africa and Southeast Asia account for more than 85% of all NE cases worldwide with regional estimates as high as 14.9 per 1000 live births [1]. Globally, over 95% of neonatal deaths and neurodevelopmental impairment occur in these regions where therapies are limited. In a retrospective cohort study in Uganda, one third of infants with NE died in the neonatal period, and of the survivors, a third developed neurodevelopmental impairment [16]. The feasibility, safety, and efficacy of HT in LMIC remains uncertain. A systematic review of seven clinical trials using various cooling devices including gel packs, water bottles, and cooled water circuits showed no treatment benefit in reducing mortality associated with NE [17]. Data for short-term and longer-term neurodevelopmental outcomes were limited. The authors were unable to conclude whether the lack of apparent treatment effects were attributed to a genuine lack of biological efficacy, related to the methodological weakness in study designs, low-level technology and core temperature instability, or suboptimal supportive care. The findings from the Hypothermia for Encephalopathy in Low and Middle-Income Countries (HELIX) study, a large multicentre, randomised controlled HT trial in south-east Asia [18] are concerning. In parallel to improving access to skilled birth attendants, good quality, timely antepartum care, and the delivery of neonatal resuscitation training programmes [19], there is an urgent unmet need to establish safe and effective therapies for babies with NE in LMIC.

Melatonin (N-acetyl-5-methoxytryptamine), an indolamine hormone has become one of the most promising neuroprotective agents for NE over the last 20 years. Its strong safety profile, ability to cross the blood brain barrier and its diverse pleiotropic properties has led to a large number of preclinical animal studies supporting its neuroprotective efficacy in reducing brain injury [20]. Whilst the preclinical data is compelling, clinical studies in infants with NE are limited [21]. Over the last decade, we have assessed the safety and therapeutic potential of melatonin to complement HT in an established, large animal piglet model of perinatal HI. The brain of the piglet, both in size and structure more closely resembles the human infant than smaller animal models [22]. The weight and size of the piglet allows standard neonatal intensive care procedures and protocols to be followed. Cerebral metabolic changes in infants can be replicated in this model [23], ensuring the translational relevance of these studies to the clinical setting. The newborn piglet model also offers the opportunity to assess in-vivo, the pharmacokinetic, physiological and systemic effects of therapeutic agents, thereby evaluating their safety, which is essential prior to clinical studies in babies.

This review summarises the main neuroprotective actions of melatonin, discusses the key findings from the University College London (UCL) neonatal piglet model of perinatal asphyxia, and explores the challenges of moving melatonin along the translational pipeline from bench to bedside to reduce the lifelong impact of NE.

## 2. The Neuroprotective Action of Melatonin for Neonatal Encephalopathy

In utero, the major source of melatonin in the developing fetus is primarily maternal. Melatonin synthesis in the pineal gland occurs in a diurnal pattern, synchronised to the light-dark cycle, reaching peak levels between 02:00 and 04:00. In response to darkness, melatonin production is triggered in the pineolycytes via a complex, autonomic neural pathway involving specialised melanopsin-containing retinal ganglion cells, the retinohypothalamic tract and the supra-chiasmatic nucleus (our biological clock) [24]. In pregnancy, melatonin is also synthesised at high levels by placental trophoblast cells and production gradually increases, particularly in the third trimester [25,26], to reach peak nocturnal levels at term [27]. Maternal melatonin readily crosses the placenta and likely drives the circadian melatonin levels we observe in the developing fetus [28,29]. Whilst the pineal gland develops early in embryonic life, the gland continues to grow in the first 2 years of life [30]. Following birth, the circadian rhythm of melatonin disappears and a period of transient melatonin deficiency ensues [31,32]. In neonatal rodents, melatonin declines rapidly in the first week of life [33]. The re-emergence of the circadian synthesis of melatonin in term infants is not observed until after 3 months of age [29,31]. The term newborn therefore has a period of relative melatonin deficiency; this deficiency might be a potential target for supplementary melatonin as a potent therapy for NE. Melatonin deficiency has been reported in intrauterine growth restriction [34] and prematurity [32], which are other potential patient groups where melatonin supplementation may be beneficial. Interestingly, Tauman et al. (2002) observed significantly lower levels of melatonin in the first weeks of life in infants with delayed psychomotor development [35]. In this review, we focus on melatonin as an acute therapy in the early hours and days after birth in term babies with NE.

### 2.1. Evolution of Brain Injury

**Figure 1:**
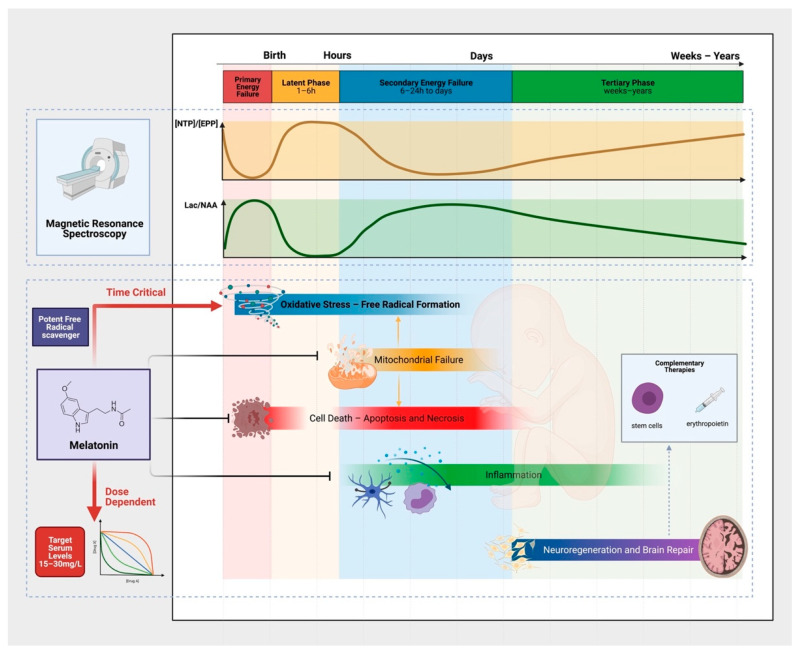
The Evolution of Brain Injury, Pathophysiology and the Neuroprotective Action of Melatonin. Characteristic phases (primary, latent, secondary and tertiary) of brain injury occurs following hypoxia-ischemia. These phases can be measured using proton (^1^H) and phosphorus magnetic resonance spectroscopy. After the primary insult and a period of recovery in the latent phase, secondary energy failure occurs (characterised by a secondary rise in Lactate to N-acetyl aspartate (Lac/NAA) peak ratio on ^1^H MRS) in parallel with the neurotoxic cascades of cellular injury. The pathological processes are multifactorial—including oxidative stress, activation of cell death pathways, neuro-inflammation, and mitochondrial failure. The neuroprotective action of melatonin is diverse. To harness its strong antioxidant properties, supra-physiological melatonin levels (15–30 mg/L) are needed early in the neurotoxic cascade. Complementary therapies including stem cells and erythropoietin targeting the tertiary phase of injury may further improve outcomes in combination with melatonin for infants with NE.

The evolution of brain injury in the first hours to days following HI occurs predictably over several phases, described elegantly with phosphorus (^31^P) and proton (^1^H) magnetic resonance spectroscopy (MRS) in newborn infants [36,37,38,39] and piglets [40,41] (Figure 1). Two seminal studies by Lorek et al. (1994) [40] and Penrice et al. (1997) [41] reproduced the sequence of events observed in newborn infants [36,37] after birth asphyxia in newborn piglets. The acute period of HI, termed the primary insult, is characterized by energy depletion, with a fall in [PCr]/[Pi] and [NTP]/[EPP], coinciding with a rise in Lactate to N-acetyl aspartate (Lac/NAA) peak ratio [41]. The metabolite changes are also associated with a fall in cerebral pH, presumed related to lactate production secondary to anaerobic metabolism [40]. Following resuscitation, a period of metabolic recovery subsequently ensues, where 31P metabolites and Lac/NAA return to baseline [40,41]. This latent phase lasts around 1–6 h (but may be longer), and is the critical window of intervention for HT. Following this period, a subsequent wave of energy failure lasting 24–48 h can be observed, described as secondary energy failure. On MRS, [NTP]/[EPP] falls and Lac/NAA rises despite adequate oxygenation, normal blood gas values and blood pressure [40,41]. The deterioration in cerebral energetics progresses further in some animals, as seen in newborn babies, whereas recovery was observed in some. The degree of secondary energy failure relates to the severity of energy depletion during the primary insult [40] and correlates with subsequent brain growth [42,43] and neurodevelopmental outcome [36,44]. The tertiary phase of injury follows, spanning weeks to years after the initial insult and characterized by persisting neuroinflammation, excitotoxity, and endogenous neuro-regeneration and repair [45]. The ability to assess changes in cerebral energy metabolism using MRS and its strong association with adverse neurodevelopmental outcomes in infants has led to interest in its use as a surrogate biomarker for NE. Following recent refinement in spectral processing, ^1^H MRS Lac/NAA peak ratio remains the most robust and accurate biomarker of early childhood outcomes in infants who are cooled [44]. Lac/NAA peak ratio is therefore one of the most important of the outcome measures in our translational model [46].

**Figure 2:**
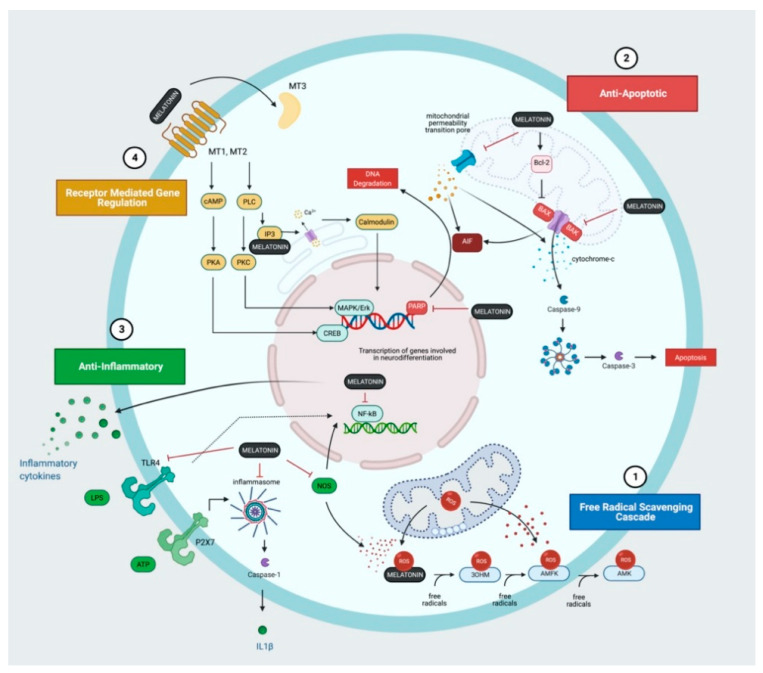
Melatonin and the Molecular Mechanisms of Action. (1) Melatonin and its metabolites; 3-hydroxymelatonin (3OHM), N1-acetyl-N2-formyl-5-methoxykynuramine (AFMK) and N1-acetyl-5-methoxykynuramine (AMK) form a potent free radical scavenging artillery that removes reactive oxygen species (ROS) and other free radicals produced following hypoxia-ischaemia. (2) Melatonin inhibits pro-apoptotic proteins (BAX), prevents the opening of the mitochondrial permeability transition pore and inhibits the poly(ADP-ribose) polymerase (PARP)-dependent cell death pathway. (3) Melatonin exhibits anti-inflammatory properties through the inhibition of nitrogen oxide synthase and Toll-like receptor 4 (TLR4) expression thereby suppressing inflammatory cytokine and chemokine production. (4) Melatonin also acts through receptors (MT1, MT2, MT3) to regulate a diverse range of downstream targets contributing to neuroprotection.

The detailed cellular and molecular mechanisms, leading to neuronal cell death and brain injury are complex and beyond the scope of this short review but are addressed by Wassink et al. (2014) [47], Gunn and Thoresen (2019) [48] and Greco et al. (2020) [49]. In this review, we will explore the key mechanisms of injury relevant to the neuroprotective action of melatonin (Figure 1 and Figure 2). Hassell et al. (2013) [50] and Tarocco et al. (2019) [51] also provide detailed review of the role of melatonin in neuroprotection for the newborn.

### 2.2. Oxidative Stress

The newborn brain is particularly vulnerable to oxidative stress compared to adults [52]. Reactive oxygen species (ROS) and other free radicals are formed early following HI [53,54]. During the primary insult, metabolites required for aerobic respiration are depleted leading to reduced ATP production and the failure of the Na^+^/K^+^ pump required to maintain the neuronal membrane resting potential and osmotic equilibrium. Membrane depolarisation leading to sodium, calcium, and water influx cause not only cytotoxic oedema, but excitotoxicity through excess release of glutamate into synaptic clefts [47]. Glutamate binds to NMDA receptors, leading to influx of Ca^2+^ into cells, activating nitric oxide synthase (NOS) and subsequent generation of nitric oxide (^•^NO) free radicals. The mitochondria are the main source of ROS (O_2_^•−^) generated, even under normal circumstances during oxidative phosphorylation. These are cleared by antioxidant enzymes such as superoxide dismutase and glutathione peroxidase. In HI conditions and during the re-oxygenation, reperfusion phase of injury, the antioxidant system is overwhelmed, leading to a release of iron and peroxide (^•^OH) ions which further contribute to oxidative damage [55]. ^•^NO diffuses readily and combines with O_2_^•−^ to generate highly destructive peroxynitrite (ONOO^−^), leading to lipid peroxidation in cell membranes, alteration of protein structure and function, and DNA damage [47,49,55]. The accumulation of ROS plays a central role in mitochondrial dysfunction, a hallmark in secondary energy failure [56]; oxidation of ETC complexes and cardiolipin peroxidation of the mitochondrial membrane result in uncoupling of oxidative phosphorylation, further ROS accumulation and failure to generate ATP. Ultimately, oxidative stress leads to the activation of cell death pathways, amplification of mitochondrial dysfunction and failure, further excitotoxicity, and neuro-inflammation contributing to the detrimental neurotoxic cascade [47,49,52]. Agents targeting the oxidative stress pathways, such as NOS inhibitors, have been exploited as neuroprotective interventions in animal studies of HI with variable success [57].

One of the remarkable neuroprotective actions of melatonin and its metabolites is its powerful and diverse antioxidant properties. Treatment with melatonin in animal studies have shown association with reduction in ^•^NO production [58] and in lipid peroxidation [59,60,61,62]. In an elegant study by Miller and colleagues (2005) [54], real-time ^•^OH production was measured in the grey and white matter of fetal sheep following umbilical cord occlusion. Maternal administration of melatonin prior to HI ameliorated ^•^OH efflux in the grey matter during primary and secondary energy failure and was associated with a reduction in lipid peroxidation on immunohistochemistry. Melatonin acts both directly, via its non-receptor mediated action as a potent free-radical scavenger and indirectly, to regulate the antioxidant defence system, promote synthesis of antioxidant enzymes, inactivate NOS, and stabilize complex I of the ETC to reduce ROS leakage [63,64]. The concept of the melatonin free-radical scavenger cascade has been adopted to describe the highly effective sequential interaction between free radicals, melatonin, and its metabolites [64]. Each melatonin molecule has the potential to scavenge up to 10 reaction oxygen or nitrogen species [64]. Melatonin is metabolised into 3-hydroxymelatonin (3OHM), N1-acetyl-N2-formyl-5-methoxykynuramine (AFMK) and N1-acetyl-5-methoxykynuramine (AMK) (Figure 2); computational modelling has shown that each have highly effective free radical scavenging capacity [65].

### 2.3. Cell Death

The mechanisms of neurodegeneration following HI is complex and have not been fully elucidated. Cell death likely manifests as a continuum between apoptosis and necrosis as observed in animal studies [66,67]. Our understanding of cell death has expanded beyond the traditional concepts of apoptosis, necrosis, and autophagy; the updated classification of cell death places a greater emphasis on molecular mechanisms rather than morphology [68]. Following HI, both the death-receptor mediated “extrinsic”, and the cytosolic, pro-apoptotic protein mediated “intrinsic” apoptotic pathways are activated. Together, these pathways lead to the interaction between the pro-apoptotic proteins, Bax and Bak, which forms mitochondrial membrane pores, resulting in the leakage of cytochrome c into the cytosol (Figure 2). An apoptosome forms, consisting of cytochrome-c, Apaf-1 and caspase 9, which activates caspase-3, the executioner of programmed cell death. The activation of the mitochondrial permeability transition pore (mPTP), a pathological membrane protein complex, also increases the permeability of the mitochondria in response to Ca^2+^ influx [69]. Melatonin’s anti-apoptotic properties include activation of Bcl-2, which inhibits Bax thereby preventing membrane permeabilization [70]. Melatonin also inhibits the opening of the mPTP via receptor-dependent [71] and receptor independent mechanisms [72,73]. There is increasing evidence that sexual dimorphism exists in the mechanisms of cell death; in females, cell death occurs primarily via a caspase-3 dependent pathway whereas in males, who are more vulnerable to oxidative stress, caspase-independent, poly(ADP-ribose) polymerase (PARP)-dependent pathways predominate [74]. The versatility of melatonin also includes down-regulation of PARP protein expression [75].

### 2.4. Neuro-Inflammation

The inflammatory process following HI includes the release of cytokines, activation of microglia, and the recruitment of peripheral immune cells [76]. In the piglet model, cytokine (IL-1α, IL-6, CXCL-8, IL-10, TNFα) expression was upregulated following HI, with and without prior inflammation-sensitisation [77] and serum IL-10 and IL-1β correlated with MRS Lac/NAA peak area ratios [78]. Clinical studies also report associations between cytokine levels in serum and CSF, severity of brain injury and neurodevelopmental outcomes in infants with NE [79,80,81]. In response to HI, the release of extracellular ATP, free radicals, and other damage associated molecular patterns (DAMPS) activate the Toll-like receptor (TLR) family (TLR2, 3 and 4 most widely studied), which triggers intracellular signalling pathways leading to NF_k_B translocation and gene transcription of inflammatory cytokines and chemokines [76]. The P2X7 receptor is also activated by extracellular ATP, resulting in microglial activation, NLRP3 inflammasome formation, caspase-1 activation and IL-1β release [76]. Rodent studies using agents targeting the inflammatory signalling cascade including IL-1 receptor inhibitor (IL1Ra) [82], candesartan, to reduce TLR2 activity [83] and TLR4 inhibitors [84] have been shown to be neuroprotective. Melatonin’s neuroprotective action extends to immunomodulation; animal studies show melatonin suppresses NOS [85] and TLR4 expression [86] thereby inhibiting NF_k_B-dependent signalling. Studies also report inhibition of NLRP3 inflammasome activation [87] although the effects on neonatal HI models remain unknown. Whilst microglia have both pro-inflammatory and reparative roles, in vitro and rodent studies suggest melatonin improves microglial survival and attenuates the pro-inflammatory polarisation of microglia [88].

### 2.5. Receptor Mediated Neuroprotection

Rodent studies using Luzindole, an antagonist against the melatonin G protein receptors MT1 and MT2, highlight a key role in the receptor-dependent action of melatonin [71]. The receptor targets of melatonin are diverse but include the widely expressed MT1 and MT2 receptors, a cytosolic MT3 binding site identified as Quinolone Oxidoreductase 2 (NQO2) and the retinoid orphan receptor (ROR) family of nuclear receptors [89] (Figure 2). The distribution of MT1 and MT2 receptors in the brain [90,91] appears to be discreet and may suggest that they have divergent functions; Klosen et al. (2019) observed MT1 expression in the suprachiasmatic nucleus and pars tuberalis in the rodent brain whereas MT2 expression was more widespread (including the forebrain, hippocampus, olfactory bulb, amygdala and superior colliculus) [91]. The downstream targets remain unclear but include gene transcription of antioxidant enzymes, regulation of synaptic plasticity, neurogenesis, and regulation of cytochrome c release in mitochondria [92,93]. The development of receptor specific ligands is needed to explore the functional differences in receptors subtypes and potential mechanisms of neuroprotection. The role of NQO2 in neuroprotection also remains controversial. Expression in the cortex, hippocampus, and amygdala [94,95] have been reported. In an adult hamster model of neurodegeneration, NQO2 activity was associated with oxidative stress and ROS induced cell death [96]. More recently, several groups also report melatonin acts via α7 nicotinic acetylcholine receptors to regulate autophagy and the inflammatory response [87,97].

## 3. Melatonin in Combination with Therapeutic Hypothermia

In high resource settings, HT is standard therapy for infants with moderate-severe NE secondary to HI, therefore the assessment of neuroprotective interventions alongside HT is necessary for such settings. Our key findings in the evaluation of melatonin in combination with HT in the piglet model of perinatal HI are summarised in Table 1.

### 3.1. Safety

Over the last decade we have shown that melatonin infusion at rates of 5–10 mg/kg/h (up to a total dose 30 mg/kg infused over 2 h) is safe and not associated with hypotension in the piglet model. As melatonin is sparingly water soluble, excipients such as ethanol are commonly used in preclinical studies [61,98,100,103] to obtain the desired highly concentrated melatonin solution for intravenous use.

Initial pilot studies using Sigma-Aldrich melatonin, diluted in ethanol (2.5% *v*/*v*) given intravenously at infusion rates ≥10 mg/kg/h was associated with hypotension requiring 0.9% saline boluses and inotropic support [98]. However, this was not observed in our most recent study using the Chiesi ethanol-free melatonin formulation given intravenously at 10 mg/kg/h [101]. It is unclear whether ethanol contributed to the observed fall in blood pressure in the pilot studies; animal studies are conflicting. Adult ovine [104] and rodent models of sepsis [105,106] report associations between high-dose 1 g/kg ethanol administration and hypotension, mediated by the upregulation of NOS, and thereby, smooth muscle relaxation, vasodilation, and reduced cardiac output. Conversely, low dose 0.1 g/kg/h ethanol infusion, which more closely resembles ethanol concentrations in our studies (~0.1 g/kg/h ethanol for 10 mg/kg/h melatonin-ethanol infusion), ameliorated septic shock in rats treated with *Escherichia coli* lipopolysaccharide [107]. Ethanol vehicle infusion in the piglet [100] and sheep models [103] of NE do not report significant blood pressure differences. However, the cardiovascular effects of ethanol require careful evaluation as following HI, haemodynamic instability is common and cerebrovascular autoregulation is disrupted.

Whilst chronic foetal alcohol exposure and the resultant neurological sequelae are well described [108], neonates receiving care in neonatal intensive care units are frequently exposed, acutely, to drugs containing ethanol excipients [109]. As part of our melatonin studies, we assessed the effects of ethanol excipient with and without melatonin in our piglet model of perinatal asphyxia. Surprisingly [100], we observed protection from low dose ethanol (0.28 g/kg) itself, observed as a reduction in TUNEL+ cell death in the white matter and improved oligodendrocyte survival. The combination of melatonin with ethanol provided significant neuroprotection and augmented HT. Indeed, neuroprotective effects of ethanol have been reported in animal models of stroke [110,111,112,113]. Possible mechanisms of neuroprotection include improved cerebral glucose metabolism through the inhibition of hyperglycolysis, leading to a reduction in lactic acid accumulation and preservation of energy (ATP) production [110]. Ethanol administration is also associated with reduction in NADPH oxidase activity, resulting in reduced ROS production [110] and in apoptotic protein expression [114].

The safety profile of melatonin doses of up to 30 mg/kg in ethanol [98,115] and ethanol-free formulations [99,101] (Chiesi Int. pat. appl. PCT/EP2018/056423) have been reassuring in our newborn piglet studies. However, there are several factors to be considered prior to the translation to first in human trials. As no licenced intravenous formulation is available on the market to date, collaboration with pharmaceutical companies is needed to expedite drug development. Melatonin is photosensitive and degrades rapidly within hours of UV-A and UV-B exposure [116], necessitating specific storage and administration requirements. Excipient agents used to solubilise melatonin should complement the neuroprotective action of melatonin and be safe for newborn use. Close attention to the pharmacokinetic (PK) profile in babies with NE is also essential. In an open-label low dose melatonin PK study in preterm infants, the half-life of elimination was prolonged (15 h) compared to adults (45–60 min) [117]. The group also noted considerable variation in drug clearance and speculated immature liver function and poor renal excretion in premature infants as contributing factors. Melatonin is metabolised by cytochrome P450 (CYP1A) enzymes in the liver and excreted in the urine. Liver and renal impairment secondary to HI may further impact melatonin elimination. Fortunately, pilot data reported that HT does not affect the PK of melatonin in term infants [118]. As discussed below, the therapeutic range of melatonin for neuroprotection from our preclinical studies is likely between 15–30 mg/L. These levels are 10,000 times higher than plasma levels in newborns at birth (typically ~80 pg/mL) [32]. The long-term consequences of supra-physiological melatonin levels remains unknown. Reassuringly, doses of up to 70 mg/kg over 24 h given to preterm infants for respiratory distress syndrome appeared safe in the short-term [119]. Jan et al. (2006) speculated that supplementary melatonin is unlikely to delay the onset of pineal melatonin production in infants as endogenous melatonin production is enhanced, rather than suppressed, in adults receiving melatonin therapy [120].

### 3.2. Efficacy and Therapeutic Levels

**Figure 3:**
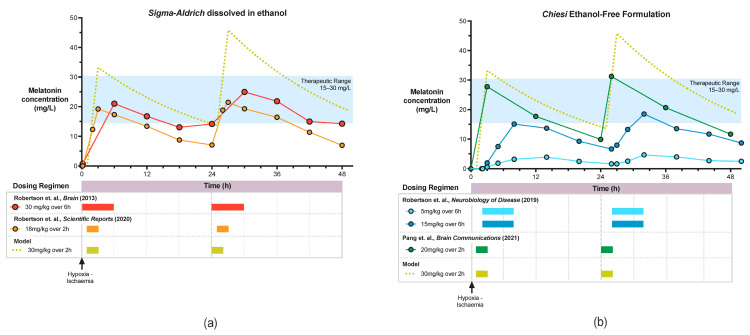
Pharmacokinetic Profile of *Sigma-Aldrich* Melatonin dissolved in ethanol (**a**) and *Chiesi* ethanol-free melatonin (**b**).

The efficacy data from four preclinical studies based on our three primary outcome measures: aEEG, ^1^H MRS Lac/NAA and immunohistochemistry are shown in Table 1. The pharmacokinetic profiles for these studies are shown in Figure 3.

In our first study, Robertson et al. (2013) [98], we observed significant augmentation of HT protection with Sigma-Aldrich melatonin/ethanol administered at 10mins after HI. Protection was based on improved aEEG recovery, improved thalamic cerebral energy metabolism (Lac/NAA and NTP/EPP) and reduction in TUNEL positive cells in 4 brain regions across the white and grey matter. Whilst isoprostane biomarkers were not significantly different, gene expression for immunomodulation towards a deactivated microglia phenotype (SOCS3, CD86 and SpK1) was observed. Melatonin levels of 17–31 mg/L were achieved using 30 mg/kg Sigma-Aldrich melatonin/ethanol, started at 10 min after HI, infused over 6h and repeated at 24 h (Figure 3). Based on these findings and in an in vitro study [102], we estimate the therapeutic range to lie between 15–30 mg/L. The widespread neuroprotection observed in this study is likely to be related to achieving therapeutic melatonin levels early on after HI, thus utilizing the free-radical scavenging properties of melatonin.

As administration of intravenous melatonin at 10 min is unlikely to be possible in the clinical setting, we explored the effect of later administration. In our subsequent study, Robertson et al., 2019 [99] administered melatonin at 2 h after HI using a slow 6 h infusion, repeated at 26 h, in conjunction with a pre-clinical dose-escalating trial using the proprietary Chiesi ethanol-free melatonin. Only partial protection was observed with the higher dose of 15 mg/kg; localised reduction in TUNEL positive cells in the sensorimotor cortex was reported but HT + melatonin did not confer improvement in aEEG background activity or cerebral energy metabolism compared to HT alone. Whilst the absence of ethanol, shown to be partially protective in Robertson et al. (2020) [100], may have contributed to the lack of treatment benefit observed. Importantly, achievement of the Cmax (16.8 mg/L) was delayed until ~8 h after HI (Figure 3). More rapid melatonin administration (18 mg/kg over 2 h) in the Robertson et al., 2020 [100] study, achieving target therapeutic levels (18.84 mg/L) earlier (within 3 h of HI), was associated with aEEG recovery from 19 h, reduction in Lac/NAA peak ratios in the white matter and thalamus and overall reduction in TUNEL positive cells compared to HT alone. Although the ethanol excipient likely added to the neuroprotective action of the melatonin solution in this study [100], our subsequent study (Pang et al. 2021) [101] using Chiesi melatonin at a higher dose of 20 mg/kg given over 2 h at 1 h after HI, repeated at 24 h and 48 h, augmented 12 h HT in all three primary outcome measures. In HT + melatonin animals, cerebral background aEEG recovery was more rapid from 25–30 h, Lac/NAA peak ratio reduced in the thalamus at 66 h, and regional reduction in TUNEL positive cells in the sensorimotor cortex was observed compared to HT + vehicle. In the absence of the confounding effects of ethanol, both studies using *Chiesi* melatonin [99,101] reported consistent localised protection in the sensorimotor cortex, a region of high metabolic activity [121] and therefore, susceptible to high levels of oxidative stress during energy failure in HI. The C_max_ achieved was higher (27.9 mg/L) and earlier (3 h) compared to the original Robertson et al., 2013 [98] study, but trough levels prior to the second dose were lower (10 mg/L vs. 17 mg/L) (Figure 3). Pharmacokinetic modelling using an escalated dose of 30 mg/kg over 2 h may increase the area under the curve within, and maintain trough levels above, the putative 15–30 mg/L therapeutic range.

### 3.3. Further Optimisation: Combination Therapies

Although melatonin holds promise, our studies suggest the neuroprotective action of melatonin is dose-dependent and time-critical; putative therapeutic levels of 15–30 mg/L within 3 h of HI are required to harness its potent antioxidant properties. This is supported by the impressive attenuation of ^•^OH efflux following umbilical cord occlusion in the grey matter of foetal sheep by early pre-insult melatonin administration [54], suggesting the antioxidative effect is likely key.

Studies of other time-critical neuroprotective agents such as Xenon, although efficacious in preclinical studies [122,123], did not translate to added treatment benefit to cooling in human trials [124,125], partly due to the delay of ~10 h before treatment could be started as babies needed to be transferred to xenon centres. Melatonin is easier to administer as an intravenous drug, mitigating unnecessary delays. However, combination therapies with several neuroprotective interventions targeting the full spectrum of the neurotoxic cascade (Figure 1) may further improve outcomes.

In our most recent study (Pang et al., 2021) [101], we assessed the safety and efficacy of combination therapy using erythropoietin (Epo) in addition to HT + melatonin (triple therapy). Epo is further along the translational pipeline, undergoing phase 3 clinical trials to assess its efficacy in combination with HT in babies with moderate to severe NE [126,127]. Its diverse mechanism of action includes not only antioxidant, anti-apoptotic, and anti-inflammatory properties, which complements melatonin, but also regenerative potential targeting the tertiary phase of injury. We observed that melatonin and Epo each augmented cooling alone, however the addition of Epo to HT + melatonin did not confer added neuroprotection in the short-term after 72 h. Unexpectedly, compared to HT + vehicle, aEEG recovery was later at 55–60 h with triple therapy and the localised reduction in TUNEL positive cells in the sensorimotor cortex observed with HT + melatonin was lost. The reason for this early negative action of Epo therapy is unclear. It is possible that delayed Epo administration may be more beneficial, and longer-term studies are needed.

There is also emerging evidence that melatonin enhances the therapeutic potential of stem cell therapies for traumatic brain injury, liver and cardiac disease, and sepsis [128]. In the piglet model of perinatal asphyxia, intranasal human mesenchymal stem cells (huMSC) augmented 12 h HT when targeted at the 24–48 h period following injury. Stem cells migrate to areas of injury and its neurotrophic response regulates cell death and neuroinflammation in the short term, and regeneration in the longer term. Interestingly, the antioxidant and anti-apoptotic properties of melatonin enhance the survival of stem cells [129]. Pre-treatment with melatonin improved the “homing ability” of stem cells to the site of injury [128]. Mesenchymal stem cells preconditioned with melatonin improved cell survival under oxidative stress and oxygen-glucose deprivation conditions in vitro [130]. In the rodent stroke model, melatonin pre-treated MSC transplantation was associated with reduction in apoptosis via an MT-receptor Erk1/2 dependent pathway [130]. The neurotrophic effects (VEGF) and neurogenesis of MSCs were enhanced with melatonin pre-treatment, resulting in improved neurobehavioral outcomes compared to animals who received untreated-MSC [130]. Studies combining the effects of early melatonin as well as “melatonin pre-treated stem cell therapies” in animal models of NE are now needed.

## 4. Melatonin as a Single Agent for the Low Resource Setting

As uncertainty remains in the safety and efficacy of HT in the low resource setting for moderate to severe NE, there is an urgent need to identify alternative, simple neuroprotective interventions. Melatonin is a promising candidate to take through the translational pipeline in such settings, given its diverse mechanisms of action and strong safety profile. Outcomes from animal studies supporting the neuroprotective effects of melatonin as a single agent for NE are compelling (Table 2). Thirteen studies [60,62,71,131,132,133,134,135,136,137,138,139,140] report improvement in histological outcomes including reduction in infarct volume, reduction in markers of apoptosis, necrosis, cell death, astrocytosis, and microgliosis. Neurobehavioral studies were also reported in 3 studies [62,132,140], observing improved cognitive performance, learning and memory, motor function, and co-ordination. Berger et al. (2019) [141] reported no overall long-term treatment effect based on brain injury volume, diffusion tensor imaging, histology and functional outcomes however the variability of injury in this model was large. Subgroup analysis revealed fewer animals with severe injury in the melatonin treated group compared to vehicle and the authors reported an absolute risk reduction for severe injury of 38.7%. The authors speculated that the dosing regimen (10 mg/kg IP given in the first day of life only; at 0 h, 6 h, and 25 h after HI) may have contributed to the lack of long-term benefit. A key limitation in the rodent studies to date is the absence of PK studies. As discussed in our piglet model, the neuroprotective effect of melatonin is likely dose dependent. The half-life of melatonin is short in rats (~20 min) and varies across animal species [142]. In the future, the inclusion of PK studies is needed to ensure optimal drug dosing and inform researchers of target therapeutic levels for future pre-clinical and human clinical trials. Care must also be taken in the choice of the excipient used to solubilise melatonin. As observed, 9 of 16 studies used 5% dimethyl sulfoxide (DMSO) (Table 2). Whilst DMSO is used in clinical practice as a cryopreservative for haematopoietic stem cell therapy [143], serious side-effects have been reported including seizures, cardiac arrest, encephalopathy, cerebral infarction, respiratory depression, severe neurotoxicity, and alternated consciousness [144]. Preclinical studies using clinically acceptable excipients for use in high-risk infants are needed.

The route of administration and dosing regimen vary across the studies. Aridas and colleagues [62] compared the efficacy of both intravenous and commercially available transdermal melatonin patches in the sheep model of perinatal asphyxia. The transdermal route is an attractive option in LMIC as patches are temperature stable; easy and quick to apply, requiring minimal training; and non-invasive, minimising infection risk. Intriguingly, the authors observed improved neurobehavioral outcomes in both administration routes and reduction in cleaved caspase-3 on immunohistochemistry. Although the transdermal route shows promise, melatonin levels were 10-fold lower compared to the intravenous route and levels were not sustained. The number of animals who received transdermal melatonin were considerably smaller, and MRS outcomes were not available. A further consideration to this route of administration is the requirement of significantly higher doses to achieve the proposed 15–30 mg/L therapeutic levels. If it is feasible to develop transdermal patches to achieve such levels, the risk of local skin reactions, which is a known side-effect of transdermal melatonin may become a concern [145].

**Table 2:** Animal studies assessing melatonin as a single agent for neonatal encephalopathy.

Study	Model	Dosing and Excipient (Pharmacokinetics If Available)	Outcomes	Other Remarks
Miller et al. (2005) [54]	Lamb model of perinatal asphyxia with umbilical cord occlusion	**Dose:** 0.5 mg/kg bolus 1 h before HI + 0.5 mg/kg/h infusion IV for 2 h **Excipient:** 1% ethanol **PK:** Cmax 12,000 pM (~2.8 μg/L) 1h after administration, 8000 pM (~1.9 μg/L) at time of HI	**Biochemical:** Completed attenuation of OH efflux in grey matter **Histology:** Reduction in 4HNE immunoreactivity in white matter and grey matter regions	
Carloni et al. (2008) [132]	Rice-Vannucci rat model	**Dose:** 15 mg/kg IP, 5 min after HI repeated at 24 and 48 h **Excipient:** 5% DMSO	**Histology:** Reduction in brain volume loss and loss of pyramidal cells in the CA1 region of the hippocampus **Neurobehavioral:** Reduced behavioural asymmetry, better performance in Morris maze	
Balduini et al. (2012) [60]	Rice-Vannucci rat model	**Dose:** 15 mg/kg IP, 5 min after HI, single dose **Excipient:** 5% DMSO	**Biochemical:** Reduction in markers of oxidative stress and nitrotyrosine expression **Histology:** Reduction in monocyte recruitment, microglial activation and astrocytosis	
Cetinkaya et al. (2011)[134]	Rice-Vannucci rat model	**Dose:** 20 mg/kg IP, given before HI, after HI and 24 h after **Excipient**: 1:10 ethanol	**Histology:** Reduction in infarct volume, reduction in TUNEL positive cells and reduction in caspase-3	Magnesium did not provide added protection in combination with melatonin
Ozyener et al. (2012) [135]	Rice-Vannucci rat model	**Dose:** 20 mg/kg IP, given before HI, after HI and 24 h after **Excipient**: 1:10 ethanol	**Histology:** Reduction in infarct volume, reduction in TUNEL positive cells in cortex and hippocampus, reduction in caspase-3	Topiramate did not provided added protection in combination with melatonin
Alonso-Alconada et al. (2012) [136]	Rice-Vannucci rat model	**Dose:** 15 mg/kg IP, 5 min after HI repeated at 24 and 48 h **Excipient**: 5% DMSO	**Histology:** Preserved neuronal survival, reduced TUNEL, preserved brain volume, reduced white matter demyelination and reduced astrocytosis	
Carloni et al. (2014) [131]	Rice-Vannucci rat model	**Dose:** 15 mg/kg IP, 5 min after HI repeated at 24 and 48 h **Excipient:** 5% DMSO	**Histology:** Reduction in brain injury volume in whole hemisphere, cortex and hippocampus	Melatonin reduces Endoplasmic Stress (epigenetic changes) and preserves SIRT1 expression
Berger et al. (2016) [146]	Rice-Vannucci rat model	**Dose:** 10 mg/kg IP immediately after HI, single dose **Excipient:** 5% DMSO	**Biochemical:** No improvement in mitochondrial metabolic function in neurons or astrocytes	DMSO may contribute to mitochondrial impairment
Hu et al. (2017) [138]	Rice-Vannucci rat model with LPS sensitisation	**Dose:** 15 mg/kg IP, 1 h prior to LPS, then daily for 1 week **Excipient:** 5% DMSO	**Histology:** melatonin improved white matter recovery and reduced blood brain barrier permeability Reduced astrocytosis and microglial activation	Melatonin suppresses TLR4 NFkB inflammatory pathway
Hu et al. (2017) [137]	Rice-Vannucci rat model	**Dose:** 15 mg/kg IP, 1 h prior to HI injury, then daily for 6 days **Excipient:** 5% DMSO	**Histology:** reduced brain tissue loss, inhibits neuronal apoptosis in cortex	
Xu et al. (2017) [139]	Rice-Vannucci rat model	**Dose:** 10 mg/kg IP, immediately after HI, single dose **Excipient:** Not reported	**Histology:** Reduction in cerebral oedema, reduction in glial cell swelling and karyopyknosis and interstitial tissue oedema. Melatonin reduces mRNA expression of oedema related proteins (AQ4, ZO-1 and occludin)	
Carloni et al. (2017) [133]	Rice-Vannucci rat model	**Dose:** 15 mg/kg IP, 5 min after HI as single dose **Excipient:** 5% DMSO	**Histology:** Reduction in necrosis and apoptosis with reduction in BAX translocation and preserved cytochrome c. Reduction in astrocytosis	Preserved SIRT1 expression, associated with autophagy activation
Sinha et al. (2018) [71]	Rice-Vannucci rat model	**Dose:** 10 mg/kg IP daily Excipient: 0.9% saline + 3% Tween	**Histology:** reduction in percentage brain loss, suppresses astrocytic (GFAP) and microglial activation (IBA-1)	Melatonin mediates effects partially through MT1 receptor. MT1 receptors downregulated following HI, upregulated with melatonin
Aridas et. Al. (2018) [62]	Lamb model of perinatal asphyxia with umbilical cord occlusion	**(6) Intravenous** Dose: ~15 mg/kg IV, started at 30 min, given as 2 hourly 5 mg boluses over 24 h **Excipient:** absolute ethanol 2 mL 0.9% saline containing 3% Tween **(2) Transdermal patches** Dose 6 × 5 mg patches (total 30 mg) applied at 30 min, replaced at 12 h **PK:** Intravenous: Cmax 150 ng/mL (~0.15 mg/L) at 4 h, Transdermal: 27 ng/mL (~0.03 mg/L) 4 h after birth, after 12 h back to baseline	**Imaging:** MRS Lac/NAA reduction **Histology:** Reduced CC3, lipid peroxidation (4HNE) and neuroinflammation (IBA-1) **Neurobehavioral:** Improved outcomes in tone, suck, standing, establishing feeds in IV and transdermal patch	Reduction in cerebrospinal lipid peroxidation and IL1β HI is associated with 3-fold increase in melatonin levels, peak with 10-fold increase at 24 h
Berger et al. (2019) [141]	Rice-Vannucci rat model	**Dose:** 15 mg/kg IP, immediately after HI, then 6h and 25 h after **Excipient:** 5% DMSO	**Imaging:** Trend towards treatment effect on Day 1 (tissue volume and ADC) but lost at day 7, 20 and 43 **Neurobehavioral:** No treatment effect **Histology:** No treatment effect	No difference in outcomes in sex
Sun et al. (2021) [140]	Rice-Vannucci rat model	**Dose:** 10 mg/kg IP immediately after HI, then every 24 h for 4 weeks **Excipient:** Not reported	**Histology:** Reduction in infarct volume **Neurobehavioral:** Improved performance in learning and memory, motor function and co-ordination and forelimb grip	*Plppr5* gene (neuronal plasticity) knockout exacerbated injury and attenuated neuroprotective effects of melatonin

4HNE = 4-hydroxynonenal, ADC = Apparent diffusion coefficient, AQ4 = Aquaporin 4, DMSO = Dimethyl Sulfoxide, GFAP = Glial fibrillary acidic protein, HI = hypoxia-ischaemia, IBA-1= Ionised calcium binding adaptor molecule 1, IP = intraperitoneal, SIRT 1 = Sirtuin 1, IV = intravenous, ZO-1 = Zonula occludens-1.

There are some limitations of current animal models for NE in the LMIC setting. Tann et al. [147] reported neonatal bacteriaemia and histological funisitis as significant independent risk factors for NE in infants in Uganda. Infants born to mothers with chorioamnionitis are at increased risk of long-term neurological sequalae [148]. In our piglet model, LPS-sensitisation prior to HI was associated with increased severity of brain injury [149]. Rodent studies suggest that the response to neuroprotective interventions, following inflammation-sensitised HI, may be pathogen specific; HT to PAM_3_CSK_4_ (gram positive) sensitised-HI was protective [150], but HT exacerbated brain injury in LPS sensitised-HI [151]. A further consideration is that current preclinical studies model an acute intrapartum HI event. However, patterns of injury on early cranial ultrasound imaging in Uganda suggest the onset of injury several hours before birth [152]. Predominant white matter injury on MRI in an observation study of NE infants in India [153] may also suggest a more chronic HI insult and/or exposure to perinatal inflammation. Preclinical studies assessing the efficacy of neuroprotective interventions in the context of perinatal inflammation and a more chronic, insidious onset of HI insults are needed.

## 5. Translating from Bench to Bedside

Although preclinical studies provide compelling evidence in the safety and efficacy of melatonin as a neuroprotective agent, clinical studies are limited. Ahmed et al. [21] reported a systematic review of clinical trials using melatonin as a single agent or in combination with HT for NE. The systematic review highlighted the lack of large, well-designed, and adequately powered randomised clinical trials in infants with NE [21] (Table 3).

To date, four of the five clinical trials [154,155,156,157] used the oral route of administration with variable doses of: 10 mg once only [156], every 2 h for 4 doses [154], or 10 mg/kg daily for 5 days [155,157] but the pharmacokinetic profiles in all studies were not reported. The oral bioavailability is less certain in newborns due to higher gastric pH, lower superior mesenteric arterial blood flow and delayed gastric emptying [158]. In adults, the absolute oral bioavailability of melatonin has been reported to be 15% [159]. Carloni et al. [160] observed serum melatonin levels of 7 mg/L were achievable with 5 mg/kg oral administration in preterm infants however, with a delay of 4 h to reach Cmax. The reduction in gastro-intestinal blood flow [161] and impaired gastrointestinal peristalsis, associated with infants with NE [162], will most likely impact the oral bioavailability of melatonin further. Given the narrow therapeutic window of melatonin, the intravenous route is the preferred route, as it provides the most predictable levels required for neuroprotection, bypassing the gastro-intestinal effects post HI and first passes metabolism in the liver. Neuroprotective agents given via the intranasal route has also received some interest [115] as it provides a more direct and rapid pathway to the brain, via the olfactory nerve and cribriform plate. Whilst this may reduce the melatonin dosing requirements and, therefore, exposure to the neurotoxic excipients, Van den Berg and colleagues [163] showed melatonin uptake in the CSF were similar between the intranasal and intravenous route. Highly concentrated melatonin formulations would be required to deliver therapeutic levels.

In the clinical melatonin trials, clinically relevant outcome measures were underreported; Aly et al. [155] observed improved survival without disability, albeit at 6 months, and Jerez-Calero et al. [164] showed improved cognitive outcomes on Bayley III neurodevelopmental assessment at 18 months in infants who received melatonin in combination with HT compared to HT alone, however the study was confounded by small sample size and a trend towards lower NE severity in the melatonin group. Consideration in future clinical design should also include surrogate biomarkers for outcomes in NE to accelerate clinical trials. ^1^H MRS Lac/NAA peak ratio has been validated in both preclinical [46] and clinical studies [44,124]. Using a Lac/NAA threshold value of 0.39, BGT Lac/NAA predicted 2-year neurodevelopment outcomes with high accuracy in infants who received HT for NE [44]. The TOBY-Xenon clinical trial, powered to detect a difference in the MRS Lac/NAA peak area ratio, recruited babies in January 2012 over 18 months, publishing in 2016 no biological effect based on difference in Lac/NAA (geometric mean difference of 1.09 (95% CI 0.9–1.32)) [125]. Follow-up neurodevelopmental data at 2–3 years of age in 2019 showed that Lac/NAA accurately predicted adverse outcomes in 96% of cases [124].

As we move towards translating melatonin from bench to bedside, several challenges remain in the drug development of suitable intravenous melatonin formulation prior to clinical trials. In general, any product used for clinical or therapeutic use should be produced to Good Manufacturing Practice (GMP); a regulation to ensure that the product is reproduceable and manufactured in a controlled environment. As the prevalence of NE is <1 in 2000 people, it is classified by the European Medicines Agency (EMA) as a rare disease, therefore eligible for Orphan Drug Designation (ODD). ODD supports pharmaceuticals by attracting investment when it is perceived that the commercial market is relatively small or the intellectual property surrounding the therapy is limited. In Europe, EMA provides access to the EMA central authorisation procedure and 10 years market exclusivity. The United States Food and Drug Administration (FDA) provide a comprehensive package including tax credits of up to 50% off the clinical drug testing cost awarded upon approval, fee waivers new drug application (NDA) or biologic licence applications (BLA) and 7 years market exclusivity.

**Table 3:** Randomised clinical trials using melatonin for neonatal encephalopathy in infants and relevant clinical outcomes.

Study	Population	Intervention	Comparison	Clinical Outcomes
Fulia et al. (2001) [154]	Single centre, Italy Diagnosis of perinatal asphyxia within 6 h, criteria not listed	N = 10 **Melatonin alone** Dose: 80 mg (10 mg 2 hourly) Route: Enteral Excipient: 1:90 ethanol:0.9% saline	N = 10 Supportive care, no HT	**Mortality:** RR 0.14 (95% CI 0.01–2.45) **Imaging:** Not reported **Neurodevelopment:** Not reported
Aly et al. (2015) [155]	Single centre, Egypt HIE: Inborn, Apgar score ≤ 3 at 5 min, BE < 12, moderate-severe NE	N = 15 **HT + Melatonin** Dose: 10 mg/kg daily for 5 days Route: Enteral Excipient: Distilled water	N = 15 HT Manual cooling with ice packs	**Mortality:** RR 0.25 (95% CI 0.03, 1.98) EEG: melatonin had fewer seizures (3/14 in HT + melatonin vs. 7/11 in HT) **Imaging:** MRI–less white matter abnormalities (0/14 in HT + melatonin vs. 4/11 in HT) **Neurodevelopment:** Improved survival without abnormalities at 6 months (10/14 in HT + melatonin vs. 3/11 in HT)
Ahmad et al. (2018) [156]	Single centre, Pakistan Any HIE *: >34 weeks gestation, based on clinical features alone * includes 12.5% mild cases	N = 40 **Melatonin alone** Dose: 10mg single dose Route: Enteral Excipient: Not reported	N = 40 Supportive care, no HT	**Mortality:** RR 0.35 (95% CI 0.14, 0.90) all cases **Imaging:** Not reported **Neurodevelopment:** Not reported
El Farargy et al. (2019) [157]	Single centre, Egypt HIE: Apgar score < 5 at 5 min, Cord pH < 7 and/or BE ≥ 12 mmol/L), and moderate NE	N = 30 **Melatonin + Magnesium Sulphate** Dose: 10 mg/kg daily for 5 days Route: Enteral Excipient: Not reported	N = 30 Melatonin alone	**Mortality:** Not reported **Imaging:** Not reported **Neurodevelopment:** Not reported
Jerez-Calero et al. (2020) [164]	Two centres, Spain HIE: ≥36 weeks gestation, severe perinatal asphyxia (Apgar < 5 at 5 min, Cord pH < 7 and/or BE ≤ −16, Moderate-severe NE (Sarnat score ≥ 6)	N = 12 **HT + Melatonin** Dose: 5 mg/kg within 6 h of birth, over 2 h, daily for 3 days Route: IV Excipient: propylene glycol + macrogol	N = 13 HT, servo-controlled, Tecotherm cooling device	**Mortality:** RR 1.0 (95% CI 0.07, 14.21) **aEEG:** No difference in background activity or seizure burden **Imaging:** No difference in MRI outcomes **Neurodevelopment:** Improved cognitive composite score (*p* = 0.05) at 18 months on Bayley III Test, no other significant differences

## 6. Conclusions

The preclinical data for melatonin as a neuroprotective agent for NE is compelling. In our piglet model, we have shown, over several studies, that melatonin is safe and augments the neuroprotective benefit of cooling. Whilst promising, to the best of our knowledge, no other in-vivo animal studies have assessed the efficacy of melatonin in combination with HT. Our studies are limited to 48–72 h in duration, and longer-term studies to assess the neurobehavioral outcomes would further support our findings. Nonetheless, a key strength to our model is the use clinical outcomes such as aEEG and magnetic resonance spectroscopy Lac/NAA which are validated outcome biomarkers to predict neurodevelopmental outcomes in babies with NE. Other preclinical studies using melatonin as a single agent support its neuroprotective efficacy in NE.

The mechanism of action of melatonin is diverse, but for optimal effect, melatonin’s antioxidant properties must be harnessed early in the neurotoxic cascade. Long-term effects of supra-physiological melatonin levels on the developing brain remains unknown. The reassuring physiological profile seen with high dose melatonin over the critical first 3 days of treatment in our pre-clinical models is encouraging. Infants with moderate to severe NE are at risk of significant mortality and morbidity; the time is now right for exploring melatonin in clinical neuroprotection trials.

Whilst the evidence for single agent melatonin therapy in preclinical animal studies of NE is valid, further studies in the context of inflammation-sensation and chronic HI are needed for the LMIC setting. The feasibility of intravenous administration and additional requirements, given the photosensitive nature of melatonin, should also be considered in this context. Robust PK studies are needed to establish the effect of HI on melatonin clearance in term infants.

The development of intravenous melatonin formulations with safe excipients (which may themselves be partially protective e.g., ethanol) for clinical use remains a key limiting step requiring the collaboration of neuroscientists, clinicians, pharmaceuticals, and research councils. In parallel, further preclinical studies are needed to identify a bundle of complementary neuroprotective agents and interventions targeting every aspect of the neurotoxic cascade to maximise the potential to change the trajectory of brain injury following perinatal HI. Agents targeting neuro-regeneration and repair (such as delayed Epo and stem-cells) to complement early melatonin administration holds promise and warrants further assessment in large recovery animal models.

## Figures and Tables

**Table 1:** Preclinical Studies Evaluating Melatonin Combined with Hypothermia for Neonatal Encephalopathy.

Study	Model	Formulation and Dosing	Pharmacokinetics	aEEG	^1^H MRS Lac/NAA	Immuno-Histochemistry	Other Outcomes
Robertson et al. (2013) [98]	Piglet model HI	Sigma-Aldrich in ethanol, 30 mg/kg over 6 h at 10 min and 24 h after HI	Cmax 21.0 mg/L at 6 h after HI Range: 17–31 mg/L	No difference	Reduction in Lac/NAA in thalamic voxel	Reduction in TUNEL+ cell death in Hip, IC, Caud, PTMN	Reduction in gene expression of CD86 and SOCS3 and increase in SphK1
Robertson et al. (2019) [99]	Piglet model HI	Chiesi ethanol-free, 5 mg/kg over 6 h at 2 h and 26 h after HI OR 15 mg/kg over 6 h at 2 h and 26 h after HI	5 mg/kg: Cmax 3.97 mg/L at 8 h after HI Range: 1–5mg/L 15 mg/kg: Cmax 16.8 mg/L at 8 h after HI Range: 7–19 mg/L	No difference	No difference	No overall TUNEL+ cell death reduction but localised reduction in sCTX	N/A
Robertson et al. (2020) [100]	Piglet model HI	Sigma-Aldrich in ethanol, 18 mg/kg over 2 h at 1 h and 25 h after HI	Cmax 18.8 mg/L at 3 h after HI Range: 7–21 mg/L	Improved aEEG from 19–24 h after HI	Reduction in Lac/NAA at 24 h and 48 h in WM and BGT voxels	Reduction in overall TUNEL+ cell count with regional reduction in pvWM and IC.	Ethanol associated with partial protection: aEEG recovery, reduction in TUNEL+ cell count
Pang et al. (2021) [101]	Piglet model HI	Chiesi ethanol-free, 20 mg/kg over 2 h at 1 h, 24 h and 48 h after HI	Cmax 27.8 mg/L at 3 h after HI Range: 10–30 mg/L	Improved aEEG from 25–30 h after HI	Reduction in Lac/NAA at 66 h in BGT voxel	No overall TUNEL+ cell count reduction but localised reduction in sCTX	Erythropoietin did not provide added neuroprotection to HT + melatonin after 72 h
Carloni et al. (2017) [102]	In vitro, Hip slice cultures	Sigma-Aldrich dissolved in 0.05% DMSO	100 μM (~25 mg/L)	N/A	N/A	Dose-dependent reduction in cell death in synergy with HT	N/A

aEEG: amplitude integrated electroencephalogram, Caud = caudate, Hip = Hippocampus, HI = Hypoxia-ischaemia, HT = therapeutic hypothermia, IC = internal capsule, Lac/NAA = lactate to N-acetylaspartate peak ratio, PTMN = putamen, pvWM = periventricular white matter, sCTX, sensorimotor cortex, TUNEL = Terminal deoxynucleotidyl transferase dUTP nick end labelling, SOCS3 = Suppressor of Cytokine Signaling 3, SphK1 = Sphingosine kinase 1.

## Data Availability

No new data were created or analysed in this study. Data sharing is not applicable to this article.
